# Remission of Persistent Negative Symptoms and Psychosocial Consequences by Combined Clozapine and Cariprazine Treatment in a Patient With Long-Standing Treatment-Resistant Schizoaffective Disorder

**DOI:** 10.3389/fpsyt.2022.887547

**Published:** 2022-05-11

**Authors:** Mats Bogren, Monica Soltesz, Stephan Hjorth

**Affiliations:** ^1^Division of Psychiatry, Department of Clinical Sciences, Lund University Hospital, Lund, Sweden; ^2^Vuxenpsykiatrimottagning Psykos, Lund, Sweden; ^3^Pharmacilitator AB, Vallda, Sweden

**Keywords:** Antipsychotic polypharmacy, negative symptoms (schizophrenia), cognitive symptoms of schizophrenia, psychosocial symptoms, quality-of-life, reward system, DA D2/D3 partial agonism

## Abstract

This patient case report describes a 45-year old white unmarried man with disability pension due to schizoaffective disorder, diagnosed at the age of 24. He lives in an apartment and has housing support. Retrospectively, the patient displayed prodromal markers of a disorder within the schizophrenia spectrum many years before the onset of frank psychosis, indeed since childhood. Over the years several symptoms and signs across schizophrenia domains have been manifest: positive, negative, cognitive, and affective, among which the negative and affective symptoms and signs were the earliest to appear. While the positive, disorganized, and catatonic symptoms responded to treatment – when duly tested and complied with – the negative and affective symptoms have been notoriously difficult to handle. We now report on the successful introduction of cariprazine (CAR) to his ongoing clozapine (CLZ) medication, the result of which has been a near-complete remission of his persistent negative and psychosocial issues. We interpret this remarkable alleviation of the patient's disease – and concomitant improvement of his quality of life – in terms of neuroreceptor target complementarity between CLZ and CAR, with particular emphasis on the contributions from the D3 and D2 receptor partial agonist components of the latter agent.

## Introduction

Schizophrenia is a devastating disorder with significant suffering and socioeconomic impact on life quality of the individuals afflicted, as well as on their families and caregivers. The lifetime prevalence is close to ~1% and therefore also linked to substantial associated health costs and significant burden to society (1). While antipsychotic medication is often helpful towards much of the positive symptom expressions of the disease, it has been estimated that about every third or fourth patient may suffer persistent residual symptoms despite adequate antipsychotic treatment (2). Among antipsychotic drug-refractory issues the negative and cognitive symptom domains appear to be particularly difficult to manage, are associated with prominent morbidity and poor functional outcome, and therefore represent major unmet medical needs in schizophrenia (3).

The Second Generation Antipsychotic (SGA) clozapine (CLZ) has become a frequently tried treatment option in treatment-refractory patients where other monotherapies have failed, triggered by the seminal paper by Kane (4) and the subsequent market reintroduction of the compound. CLZ is also endorsed as a third-line option in many schizophrenia Treatment Guidelines (5). While often efficacious, CLZ (monotherapy) treatment may nonetheless leave some patients with only partial resolution, hence left in a state with residual symptom issues – particularly regarding negative and cognitive traits. Indeed, a meta-analysis suggests but a ~40% response rate to CLZ in treatment-resistant patients (6). Moreover, CLZ carries many side effect liability risks, e.g., metabolic effects, hypersalivation, constipation and enuresis. The recently launched Third Generation Antipsychotic (TGA) partial dopamine agonist agent cariprazine (CAR) has been shown to be an effective ‘broad-spectrum’ option in the treatment of schizophrenia (3, 7, 8). Of particular note, this agent has proven effective not only to manage positive symptoms, but also to bring about clearcut improvement (superior to risperidone) in patients with long-standing predominant primary negative symptoms in a carefully controlled randomized double-blind study (9). This action of CAR has been attributed to its very high affinity and partial DA receptor agonism at the D2 and, in particular, D3, receptors, hence distinguishing CAR from older agents from the First and Second Generation Antipsychotic drug classes (FGA and SGA, respectively), including CLZ.

## Case Presentation

Below we detail the disease-relevant background history of our patient (overview in Table 1), followed by a section describing the clinical changes upon the recent introduction of CAR to his antipsychotic treatment regime (CLZ and valproate).

**Table 1:** Timeline of patient biographic and medical events until 37 years of age.

**Age**	**Events**	**Comments**
*0-2 y*	Birth and early development	48 hour long delivery. Strongly icteric at birth. Late in reaching developmental milestones: walking, potty training.
*3*–*5 y*	Preschool years	Quiet, shy, afraid of knives, spiders and new things. Orderly.
*6*–*18 y*	School years	When starting school the patient complained about one foot being malformed (upon examination the foot was normal). Socially uncomfortable, few friends, conscientious. Lack of joy. At 11 the patient unexpectedly and suddenly became agitated with pressured speech and throwing things around. Graduated from high school with good grades.
*19*–*21 y*	University studies	Dropped out after 2 years. Described feelings of indifference, lack of motivation, and difficulties in concentrating and remembering.
*21 y*	Started psychotherapy	The psychotherapy was initiated by the patient's mother motivated by her observation that the patient had become “like a zombie”: increasingly withdrawn and passive, while at the same time obsessively controlling things and ruminating over being physically ill. However, the psychotherapy was terminated as the patient's condition worsened during the treatment and the patient was referred to the psychiatric services.
*21*–*22 y*	Initiated contact at the open psychiatric care clinic	The patient received 3 months of citalopram- and open day care treatment, subsequently followed by a new trial of psychotherapy focusing on low self-esteem. The treatment had no positive effect. Once again, the psychotherapy had to be terminated due to worsening of the patient during treatment, including aggravation of depressive mood, obsessivity and hypochondriacal concerns about cancer, now alternating with 6–12 h long hyperactive and elated episodes.
*23 y*	Started work as a cleaner	Quit the job due to lack of energy.
	Started treatment with nefazodone	
	Suicide attempt	One week after nefazodone initiation the patient intoxicated himself with zolpidem (was found by the mother).
	First period of psychiatric inpatient care	Following the suicide attempt the patient was admitted for 12 days of psychiatric inpatient care. During the stay the patient – who was reported to be passive and showing no spontaneous speech – was diagnosed with bipolar depression. A psychometric evaluation demonstrated evenly distributed cognitive functions within the normal range (IQ: 108). The patient was prescribed to continue the nefazodone treatment and was discharged with a plan of continued open psychiatric care and treatment.
	Started treatment with venlafaxine at the open care clinic	Due to absence of effect after about 2 months of treatment nefazodone was switched to venlafaxine.
	Another suicide attempt	About 2 weeks after the initiation of venlafaxine treatment the patient intoxicated himself with caffeine tablets.
	Second period of psychiatric inpatient care	After the second suicide attempt the patient was admitted for renewed psychiatric evaluation and treatment for 4 months. No signs of positive psychotic symptoms were observed, but as the patient – apart from the brief episodes of hyperactivity and elation that continued to appear – was fundamentally withdrawn, apathetic and showed signs of affective blunting and anhedonia schizophrenia was suggested. Treatment with risperidone was initiated but had to be discontinued because the patient did not accept it. The patient was discharged.
*24 y*	Continued contact at the open psychiatric care clinic	After discharge the patient had contact with the day care unit and for 5 months he accepted treatment with lithium. The status of the patient did not change during the lithium treatment: he continued to appear depressive and apathetic with blunted affect, occasionally interrupted by brief hypomania-like episodes. Subsequently, the patient stopped attending the open psychiatric clinic and withdrew the lithium treatment with the motivation that he did not want to be dependent on pills. He denied side effects.
	Started another job as a cleaner	After about 8 months the patient was fired because of “inadequate behaviour”.
	Resumed contact with the open psychiatric clinic, including the day care unit	When the patient came back to the day care unit he appeared unconcentrated, absent minded, sometimes inappropriately laughing and expressing vague ideas of reference and feelings of being influenced – perhaps by God – via the radio and television. The patient had lost about 10 kg of weight. His apartment was found to be completely disorganized.
	Third period of psychiatric inpatient care	The patient was hospitalized by force for about 6 months. During the hospitalization the patient made stereotyped movements with his hands and arms, and reported on experiencing chaotically changing feelings – in stark contrast to earlier emotional numbness – which made thinking unnecessary, depersonalization, derealization as well as telepathic and other nonverbal messages from people, including celebrities. He also described having auditory hallucinations with commenting, imperative and discussing voices. Schizoaffective disorder was diagnosed
*25 y*	Treatment with risperidone, lithium and valproate was started during the forced hospitalisation	Under treatment with risperidone, lithium and valproate productive psychotic symptoms become reduced, but the patient was indifferent, anhedonic and apathetic. When venlafaxine and reboxetine, respectively, was added, and when risperidone was switched to ziprasidone, positive psychotic symptoms reemerged. With continued risperidone-, lithium- and valproate treatment psychosis attenuated slowly. The patient was discharged from forced inpatient care to the open psychiatric clinic. Indifference and emotional numbness remained.
*26 y*	The patient stopped taking the prescribed medication	Within weeks after discontinuing the medication the patient suffered a psychotic relapse with similar symptoms as previously.
	Fourth period of psychiatric inpatient care	The patient was hospitalized by force for a second time and during a 1 month stay the treatment was reinstated, after which the patient was discharged for continued open care. Shortly after discharge the patient caused a fire and indoor flooding in his apartment (when he noticed the fire he opened all the water taps) and was brought by the police to the psychiatric emergency unit.
	Fifth period of psychiatric inpatient care	Another period of forced psychiatric care ensued for 7 months. The psychotic symptoms were now even more difficult to treat than before; risperidone and perphenazine yielded unsatisfactory results. Finally positive psychotic symptoms responded to treatment with clozapine and valproate, although negative symptoms remained prominent and unchanged (PANSS positive symptom score was reduced from 24 to 5, while PANSS negative symptom score only dropped from 26 to 21). The patient was discharged to live in an apartment with housing support.
*27*–*37 y*	For about 10 years the patient took part in several rehabilitation trials, which all failed. At age 37 the patient received disability pension	The patient lives more or less isolated in his apartment, reluctant to accept housing support. He dreads becoming psychotic again and does not want to change his medication. He suffers no relapse of psychosis, but negative symptoms and side effects from the treatment are prominent.

### Background History

Varying expressions of mental health issues were identified in the family history of our patient. Thus, the maternal grandfather and a maternal aunt suffered from compulsive controlling behaviour. The grandfather, who was an introvert person, committed suicide. The father of the patient was described as aloof, and a paternal aunt suffered from depressions.

The early years of the patient were characterized by introversion, anxiety proneness and orderliness. During adolescence he had few friends, but did well in school, being meticulous about his studies. Premorbidly, the patient was disposed to social anxiety, fear of change, with hypochondriacal sensations and thoughts; according to his mother, from 14 years of age he rarely if ever showed any signs of joy. Nevertheless, it was some time after the patient started university studies that his mental health started to seriously deteriorate. He lost drive, and found it harder and harder to focus on and remember things. Moreover, his habitual orderliness and health worries grew into obsessiveness and compulsiveness, and intense hypochondriacal fears. He became nonreactive to his environment. By the time the patient was 21 his mother was seriously concerned and arranged for him to see a psychotherapist. However, the therapy did not work – he continued to deteriorate and was transferred to psychiatry. For the subsequent 2 years the patient entered into fruitless treatment trials with citalopram, nefazodone, venlafaxine and psychiatric day care. Another round of psychotherapy was also undertaken. A detailed overview of the pharmacological treatment history is found in Table 2.

**Table 2:** Overview of pharmacological treatment history^*^.

**Date (age)**	**Treatment**	**Comment**	**CGI-S**
1997 (21y)	Citalopram, dose unknown.	No effect. Terminated after 3 months treatment. Passive, indifferent, depressive, obsessive, hypochondriacal.	4
Jan 1998 (22y)	Nefazodone 100 mg. Zolpidem 7.5 mg.	One week after introduction of Nefazodone, suicide attempt through overdose of Zolpidem. No information about adherence, or if the patient withdrew Nefazodone prior to the suicide attempt. 12 days of psychiatric inpatient care followed.	
Jan 1998 (22y)	Nefazodone raised to 200 mg.	During inpatient period the patient was withdrawn without spontaneous speech. No effect from Nefazodone treatment.	5
Feb 1998 (22y)	Nefazodone raised to 400 mg.	No effect. Nefazodone was terminated after 2 months in conjunction with switch to Venlafaxine.	
Mar 1998 (22y)	Venlafaxine up to 150 mg.	Two weeks after introducing Venlafaxine, suicide attempt through overdose of caffeine tablets. Adherence unknown, or whether the patient withdrew Venlafaxine prior to the suicide attempt. The patient was hospitalized 4 months. Venlafaxine terminated.	
Apr 1998 (22y)	Risperidone 3 mg.	During the inpatient period the patient was withdrawn, apathetic, blunted and anhedonic, which evoked suspicion of schizophrenia despite lack of signs of psychosis. After 4 months the patient refused to continue the Risperidone treatment and was discharged to day care without medication. No significant treatment effect was observed.	5
Nov 1998 (23y)	Lithium up to 6 x 42 mg.	Accepted Lithium monotherapy for 5 months, but then refused. No significant treatment effect was observed.	5
Apr 1999 (23y)	No pharmacological treatment.	No psychiatric contact	
Nov 1999 (24y)	No pharmacological treatment.	Resumed contact with day care. Was absent-minded, disorganized, occasionally giggling and expressing ideas of reference/influence. The condition worsened. Eventually hospitalized 5 months.	6
Feb 2000 (24y)	Risperidone 4 mg. Lithium, 6 x 42 mg.	Psychosis considerably reduced after reintroduction of Risperidone combined with Lithium, but feelings of emptiness/numbness remained along with apathy and blunting. Erratic adherence to Risperidone/Lithium treatment after discharge.	5
Nov 2000 (25y)	Venlafaxine up to 150 mg added to Risperidone/Lithium.	Psychotic symptoms reappeared. Hospitalized for a month.	6
Dec 2000 (25y)		Venlafaxine terminated. Continued Risperidone/Lithium.	5
Feb 2001 (25y)		Plasma-Lithium 0.82 mmol/L	
Mar 2001 (25y)	Reboxetine up to 6 mg added to Risperidone/Lithium.	Continued Reboxetine for 2 months. No effect on depressive or negative symptoms. Was briefly hospitalized. After discharge psychotic symptoms reappeared and Reboxetine was terminated.	5
May 2001 (25y)	Risperidone tapered and switched to Ziprasidone up to 80 mg. Continued Lithium.	After introduction of Ziprasidone hypomania developed and psychosis intensified. Hospitalized 6 weeks.	6
	Valproate up to 600 mg added to Ziprasidone/Lithium.	Improved, but withdrew the treatment upon discharge. About a month later overt psychosis developed. Forcibly admitted.	6
Sep 2001 (26y)	Risperidone up to 6 mg and Lithium 6 x 42 mg was reinstated.	Psychosis started to slowly attenuate but did not go into remission. Emptiness, numbness, apathy, and blunting remained. After discharge the patient withdrew treatment and did not attend day care as recommended. Decompensated quickly, caused fire in his apartment, and was forcibly admitted again. Was admitted nearly 2 years, though with extended permission periods from the ward towards the end.	6
Oct 2001 (26y)	Lithium 6 x 42 mg reinstated. Lithium combined with Risperidone 6 mg for 2 months, followed by tapering Risperidone and switch to Perphenazine (up to 24 mg) for 1 month, after which also Perphenazine was tapered.	PANSS Oct 2001: Total 91, positive 24, negative 26. Effect of Lithium/Risperidone and Lithium/Perphenazine, respectively, was unsatisfactory. While tapering Perphenazine, Clozapine was introduced.	5
Dec 2001 (26y)	Clozapine successively raised to 500 mg during 2 months. Lithium was terminated and Clozapine continued as monotherapy.	PANSS Feb 2002: Total 94, positive 19, negative 34.	5
May 2002 (26y)		Positive psychotic symptoms attenuated slowly, but negative symptoms remained. PANSS: Total 48, positive 5, negative 21. Moved to open ward.	4
July 2002 (26y)	Clozapine raised to 600 mg.		
Sep 2002 (27y)	Valproate up to 1200 mg added.	Valproate introduced to reduce risk of relapse into mania.	
Sep 2003 (28y)		After managing gradually more extended periods of permission from the hospital the patient was discharged. P-Clozapine: 1765 nmol/L. P-Valproate: 488 micromol/L.	4
Apr 2004 (28y)	Clozapine raised to 650 mg.		
Feb 2005 (29y)	Clozapine decreased to 600 mg.		
Mar 2005 (29y)		P-Clozapine: 404 nmol/L. Reason for low level unknown. Non-adherence? P-Valproate: 379 micromol/L.	
Apr 2005 (29y)	Citalopram up to 90 mg added.	Marginal effect of Citalopram on anhedonia, obsessions-compulsions or phobias. P-Clozapine: 1,000 nmol/L.	4
Feb 2006 (30y)		P-Clozapine: 1,880 nmol/L. P-Citalopram 741 nmol/L. P-Valproate: 391 micromol/L.	
Mar 2006 (30y)	Desmopressin 0,2 mg. Oxybutynin up to 5 mg + 2.5 mg + 15 mg.	Nocturnal enuresis issues. Desmopressin tried, but withdrawn after 2 weeks due to lack of effect. Switched to Oxybutynin. Oxybutynin yielded some, but insufficient, effect on cholinergic complications. After a year the patient withdrew it.	
May 2007 (31y)	Clozapine decreased to 500 mg.	P-Clozapine: >4,000 nmol/L. Reason for high level unknown. No trough concentration? Increased caffeine consumption? The patient had not changed his smoking habits.	
Apr 2008 (32y)	Clozapine decreased to 450 mg.		
Dec 2010 (35y)		P-Valproate: 651 micromol/L.	
Feb 2013 (37y)	Citalopram tapered and switched to Sertraline up to 200 mg.	No further effect on anhedonia, obsessions-compulsions or phobias was observed.	4
Jul 2014 (38y)		Quit smoking.	
Sep 2014 (39y)		P-Clozapine: 2,850 nmol/L. Remained non-smoking.	
Jun 2015 (39y)		P-Clozapine: 3,410 nmol/L. Remained non-smoking.	
Jun 2019 (43y)	Cariprazine up to 4.5 mg added to Clozapine 450 mg/Valproate 1,200 mg/Sertraline 200 mg	About 2 months after introduction of Cariprazine the patient describes “a warm feeling in the body” and wants to plan and fix things. He is well groomed and chatty. Another month later the patient gets up at 7 am and frequently leaves his apartment. Upon contact he shows normal reactivity.	3
Oct 2019 (44y)	Clozapine decreased to 425 mg.		
Mar 2020 (44y)	Clozapine decreased to 400 mg.		
May 2020 (44y)	Clozapine decreased to 375 mg. Cariprazine raised to 6 mg.		
Sep 2020 (45y)	Clozapine decreased to 350 mg.	Time required for compulsive checks has decreased from 2 h to 30 mins per day.	2
Dec 2020 (45y)	Clozapine decreased to 325 mg.		
Mar 2021 (45y)	Clozapine decreased to 300 mg.	P-Clozapine: 1,470 nmol/L.	
May 2021 (45y)	Clozapine decreased to 275 mg.	May 2021 medication Cariprazine 6 mg/Clozapine 275 mg/Valproate 1,200 mg/Sertraline 200 mg.	

* *Periodically the patient has also used alimemazine, propiomazine and levomepromazine, occasionally benzodiazepines in small doses. Due to his fear of becoming addicted, none of these treatments have been used since 2014. In periods he has also received physiotherapeutic treatment*.

*CGI-S, Clinical Global Impression Severity score*.

Following the introduction of nefazodone and venlafaxine, respectively, the patient attempted to commit suicide twice, which prompted periods of psychiatric inpatient care. During the second round of psychotherapy the lowered mood intensified, which however – which was new – was briefly interrupted by hour-long hypomania-like episodes. The patient was diagnosed with bipolar depression. However, it was at this point also speculated that his clinical presentation (with prominent affective blunting, alogia, apathy and anhedonia), despite the hypomania-like episodes and absence of psychosis, could actually be signs of schizophrenia. However, the patient did not accept treatment with an antipsychotic, which was suggested. He did accept a treatment trial with lithium though, which, unfortunately, had no positive effect (Table 2). About a year later, at age 24, the patient – during a period of work as a cleaner – decompensated severely and displayed overt psychosis. At this time, a diagnosis within the schizophrenia spectrum was rather obvious, as was the need for antipsychotic treatment. However, the patient was not cooperating adequately and was committed to psychiatric inpatient care at three separate occasions after having stopped his medication (risperidone plus lithium, ziprasidone plus lithium and valproate, and risperidone plus lithium, respectively; Table 2) during the following 3 years. During these admission episodes the patient was intensely psychotic and disorganized, and his response to antipsychotic treatment weakened more and more with each new episode. In the end, after also having tried perphenazine plus lithium, combined treatment with CLZ and valproate reduced the positive psychotic symptoms to a minimum, but notably negative symptoms persisted and dominated the picture (Table 2). The patient attained retrospective insight, and has since been very careful not to change his medication out of fear of becoming psychotic again. While the aforementioned antipsychotic regimen thus worked to prevent more psychotic relapses, it came at a price. The treatment is accompanied by side effects (i.e., hypersalivation and nocturnal enuresis) and has not accomplished alleviation of the negative symptoms: hedonic deficiency, weak social-, self care- and volitional drive, marked taciturnity and hyporeactive affectivity.

Rehabilitation efforts between age 27 and 37 failed, probably partly because of the patient's evasive and introverted attitude, and partly because of a general sense of social defeat, social stigma, and fear of failure. At 37 the patient accepted disability pension.

### Clinical Course Since the Initiation of Cariprazine Treatment

For many years following his disability pension, the patient lived more or less in isolation. Apart from his mother and housing supporters – whom he was not keen on seeing – he met very few people. His life was dominated by negative symptoms, anxiety, compulsive checks, and side effects from the drug treatment. He suffered from a reduced ability to translate will and wishes into action. He often stayed in bed, but h were also spent checking the stove and water taps, triggered by a fear of fire and indoor flooding. Because of such obsessive thoughts the patient seldom left his apartment. The personal hygiene was neglected, he seldom shaved, and the apartment was filled with unwashed dishes and unread mail. The housing supporters offered help but were often rejected. The patient often missed appointments – e.g., at the psychiatric open clinic - despite reminder calls, and he did not accept home visits. He occasionally heard voices, which made him scared of becoming uncontrollably psychotic again. Because of this he did not for a long time want to lower the dose of CLZ in spite of the difficult-to-tolerate cholinergic system side effects (hypersalivation, constipation and nocturnal enuresis). Treatment with SSRI:s (citalopram, sertraline) only minimally affected anhedonia, obsessive-compulsive symptoms and phobias. He refused treatment with aripiprazole, which was suggested. Eventually, after repeated motivation, the patient agreed on lowering the dose of CLZ by small steps from 600 mg daily (age 29) to 450 mg daily (age 34). Side effects decreased somewhat, but did not cease, and the patient's condition remained virtually unchanged.

At age 44 (year 2019) – after recurrent persuasion – the patient accepted a treatment trial with CAR. CAR treatment was started with 1.5 mg per day for 4 days, followed by 3 mg per day for 12 days, after which the dose was raised to 4.5 mg per day [slow titration strategy (10)]. The patient reported no side effects and no adverse reactions were observed. 2 months after the initiation of CAR the patient – whom had now shaved off his long beard – was talkative and described that he had “*a warm feeling in the body*”. He also said that he wanted to fix certain things: mend the bicycle and buy a new mobile phone (the old one had been broken for 3 years). Another 2 months on, the patient – on his own initiative – suggested that the dose of CLZ should be lowered more to reduce side effects: the dose was lowered to 425 mg per day. Another 5 months later the patient reported that he was feeling happy and alert, and that he had started to get up at seven o'clock in the morning (for many years he had used to sleep or stay in bed until the afternoon). He also described that he had started to go out. The patient reported this with a normal speech flow and reactivity under the conversation. The dose of CLZ was lowered to 400 mg per day. Subsequently the patient made some friends and continued to see them; the bike was fixed and a new phone was purchased. About 11 months after the initiation of CAR treatment the dose was increased from 4.5 mg to 6 mg per day. Some 4 months thereafter the patient reports that he has stopped obsessively controlling the lock of his door and that other controls are less time consuming: they have been reduced from about 2 hours to about 30 mins per day. For the next 8 months the dose of CLZ is further reduced to 275 mg per day. Following the CLZ reduction constipation disappeared, and hypersalivation and nocturnal enuresis were significantly, although not completely, reduced. (While the plasma level of CLZ at this dose is regrettably not available, 300 mg/d resulted in 1470 nmol/L; see, Table 2). The patient had never had any metabolic complications from CLZ. No signs of psychotic relapse or exacerbation of other symptoms have been noticed or reported. Since the initiation of CAR (and CLZ dose reduction) the patient has started to accept the housing supporters, despite not really needing them as much as before as he has often made the dishes and cleaned the apartment by himself. Likewise, the need to be reminded of things had disappeared. The patient's mother has reported that the patient has become more open and active in their contact. Another striking observation is that the patient has become increasingly social: he regularly sees his friends and has resumed contact with his father, whom he hadn't seen for many years. In conclusion, the life quality of the patient has improved notably. Moreover, as seen in Table 2, his CGI-S scores (11, 12) that had recurrently ranged between moderately and severely ill (scores 4–6) across the years ever since 1997, following the introduction of CAR in 2019 dropped to mildly to minimally ill (scores 2–3).

## Discussion

This case report describes a 45-year old male with an extensive history of psychiatric disease afflictions (social anxiety, phobias, obsessive-compulsiveness, hypochondriacal fears, attentional deficits, affective blunting, hypohedonia, abulia, suicidal attempts, bipolar depression, hypomania etc.) before finally presenting with overt psychosis at the age of 24 (including ideas of reference and influence, auditory hallucinations, emotional turmoil, stereotyped psychomotor signs and disorganisation). Following several years of at best partially successful antipsychotic treatments and prominent sustained negative and cognitive symptoms, the introduction 2 years ago of CAR alongside his ongoing CLZ treatment has turned his clinical picture into near-complete remission.

The treatment record of our patient up to the psychosis debut had included fruitless trial attempts with lithium, citalopram, nefazodone, venlafaxine, as well as unsuccessful psychiatric day care and psychotherapeutic approaches. Following the schizoaffective disorder diagnosis, several efforts to find an efficacious antipsychotic treatment regimen were also instituted: risperidone, ziprasidone and perphenazine in combination with lithium (Table 2), but with inadequate clinical efficacy which may have been due to a lack of compliance from the patient. The patient did not accept the use of long-acting injection antipsychotics. Although eventually a schedule based on CLZ and valproate was found to successfully alleviate his positive symptoms, the prominent affective blunting, negative and cognitive symptomatology (presenting already prior to his *bona fide* diagnosis of schizophrenia) continued to constitute a dominating part of his issues, along with anankastic behaviours. Retrospectively, the disease history of our patient (extending into childhood/adolescence; see, Table 1) may also be seen to be consistent with the neuro/sociodevelopmental theories of schizophrenia (13, 14).

We decided to introduce CAR in the treatment of our patient based on the consideration that it would furnish a complementary neuroreceptor profile to that of CLZ, with net potential benefits both regarding efficacy and adverse event readouts (15–17). Thus, CAR (monotherapy) has been shown to greatly improve predominant primary negative symptoms in patients with schizophrenia (9, 18), including marked enhancements also in the “Personal & Social Performance” domain (9), as well as in the recovery of attentional cognitive processes (19). Further, CAR also appears beneficial from a relapse prevention point-of-view (20, 21), which may be viewed as advantageous in the present context as the patient was afraid of relapsing into psychosis upon lowering the dose of CLZ. We also hoped that the addition of CAR would enable a further dose reduction of CLZ to reduce CLZ-induced side effects. Interestingly, a case series published during the review process of the current manuscript describes the successful treatment of negative symptoms by addition of CAR to CLZ in five treatment-resistant schizophrenic patients, adding further support for the potential clinical usefulness of this combination (22).

**Figure 1:**
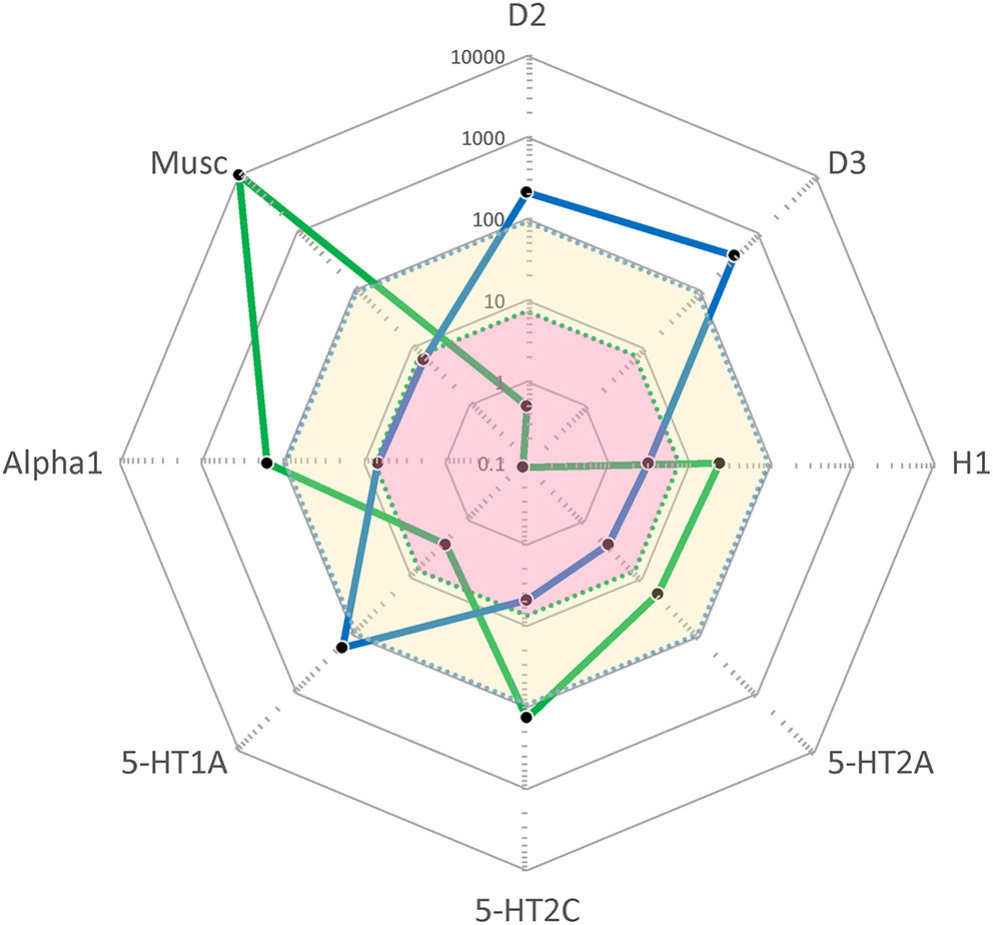
“Cobweb” depiction of Clozapine (CLZ; blue) and Cariprazine (CAR; green) target profiles overlaid on the *free* (unbound) steady-state plasma concentrations (nmol/L) of these antipsychotics at average clinical dosage (CLZ: yellow area; CAR: pink area). Black dots correspond to drug affinities reported in the literature (in nM) for the targets labeled on the edges of the cobweb; the closer to the center, the higher affinity for the target in question. (For further detail, see Hjorth ^16^). D2, dopamine D2 receptor; D3, dopamine D3 receptor; H1, histamine H1 receptor; 5-HT2A, serotonin 2A receptor; 5-HT2C, serotonin 2C receptor; 5-HT1A, serotonin 1A receptor; Alpha1, α1-adrenoceptor; Musc, ACh muscarinic receptors.

As seen in Figure 1, CLZ and CAR display entirely different target profile “fingerprints”. Thus, CLZ is an antagonist with quite poor affinity for the DA D2 (and D3) receptor but carries high affinities for H1, 5-HT2A, 5-HT2C, alpha1, and muscarinic sites. For comparison, CAR is a high-affinity D2/D3/5-HT1A partial agonist with preference for the D3 receptors, but lacking appreciable affinity for the set of sites for which CLZ displays high potency. There is complementarity between the two antipsychotics also from a pharmacokinetic perspective; at steady-state CLZ has a half-life of ~12–14 h while CAR [together with its active metabolite di-desmethyl-CAR (23)] has an effective half-life of ~7 days (20) and may thus be viewed as a “long-acting oral” medication.

Upon reflection, the history of our patient may install some hope regarding the possibility to treat stationary cases with refractory schizophrenia. In particular, one may speculate that the primary or enduring negative and cognitive deficit states often seen in schizophrenia are not necessarily the result of irreversible neurodegeneration, but may rather represent dynamic brain states that are still amenable to intervention and change. In part the negative syndrome might be driven by a motivational-volitional disorder that hinges back to a disturbance of the reward system of the brain (24). The potential of CAR to improve predominant primary negative symptoms (9), along with its pharmacological ability to modulate the transmission of dopamine via D3/D2 receptor partial agonism within the mesolimbic and mesocortical systems, is congruent with such a hypothesis.

## Strengths and Limitations

Obviously, the main limitation of the work is that it concerns the description of a single patient case, and therefore cannot be immediately generalized to a wider patient population. Apart from the use of PANSS when CLZ was introduced, the symptomatic presentation (its positive, negative and cognitive, as well as social and quality-of-life dimensions) were not consistently assessed with validated scales during the course of the illness, e.g., with formal assessments of negative symptoms using the SANS scale (25). This of course limits a stricter objective quantification of the treatment response. This said, the global illness severity has been regularly assessed with the CGI-S instrument (11, 12) already from the outset of the patient's contact with psychiatry, and confirms the striking improvement experienced after the addition of CAR to his ongoing antipsychotic treatment with CLZ (Table 2). In addition, the extended and detailed account of the patient's disease journey, encompassing diagnosis and close management follow-up from clinical as well as pharmacological viewpoints is a clearcut strength, particularly as two of the authors (MB & MS) have been able to regularly monitor the clinical course of this patient across several years.

## Conclusion

CAR is a promising new agent in the treatment of schizophrenia spectrum conditions. Besides having antipsychotic properties, CAR can potentially alleviate predominant primary negative symptoms, as well as social and cognitive deficits, all of which represent still unmet treatment needs in schizophrenia. Due to its unique target profile fingerprint–including dopamine partial agonism and D3 over D2 receptor preference–CAR may complement the pharmacological and clinical profile of CLZ and modulate dopamine transmission within brain systems that are important for reward and cognition. We report on a case with a long-standing treatment resistant schizoaffective disorder in which treatment with CLZ and valproate had reduced the positive but not the negative and psychosocial symptoms, but where following addition of CAR a remarkable improvement took place. Severe, functionally debilitating, negative and psychosocial symptoms, including anhedonia, abulia, affective blunting, alogia and social amotivation and withdrawal, almost disappeared, and obsessive symptoms concomitantly decreased, overall resulting in an amazing quality-of-life enhancement. Moreover, after the introduction of CAR to the ongoing CLZ treatment, the dose of the latter could be reduced which considerably reduced side effects. We hypothesise that the pharmacological complementarity between CAR and CLZ underlies this action, where CAR's high affinity partial agonism at D2/D3/5-HT1A receptors and preference for the D3 receptor, supplements the target impact of CLZ, thereby resulting in an efficacious and clinically beneficial neuroreceptor/circuit interaction outcome. Needless to say, controlled studies of adequate size and duration are warranted to further substantiate the findings in this case report.

## Data Availability Statement

The datasets presented in this article are not readily available because the data are extracted from a patient medical journal and is thus personally confidential within the framework of the medical professionals involved in his treatment. Requests to access the datasets should be directed to mats.m.bogren@skane.se

## Ethics Statement

Ethical review and approval was not required for the study on human participants in accordance with the local legislation and institutional requirements. The patients/participants provided their written informed consent to participate in this study. Written informed consent was obtained from the individual(s) for the publication of any potentially identifiable images or data included in this article.

## Author Contributions

MB and MS oversaw the patient's clinical management and MB wrote the original draft. MB, MS, and SH conceptualized and researched the subject, conceptualized, reviewed, and edited the manuscript. All authors contributed to the article and approved the submitted version.

## Conflict of Interest

MB and SH have received lecturing and advisory board honoraria from Recordati. The writing was in part supported by Recordati, but the company had no influence on data collection, analysis, content, or interpretations presented; the authors alone are responsible for the content and writing of the paper. Recordati also provided funds for the open access publication fees. The remaining author declares that the research was conducted in the absence of any commercial or financial relationships that could be construed as a potential conflict of interest.

## Publisher's Note

All claims expressed in this article are solely those of the authors and do not necessarily represent those of their affiliated organizations, or those of the publisher, the editors and the reviewers. Any product that may be evaluated in this article, or claim that may be made by its manufacturer, is not guaranteed or endorsed by the publisher.
